# Cerebral Ischemia Changed the Effect of Metabosensitive Muscle Afferents on Somatic Reflex Without Affecting Thalamic Activity

**DOI:** 10.3389/fphys.2018.00638

**Published:** 2018-05-29

**Authors:** Caroline Pin-Barre, Christophe Pellegrino, Frédéric Laurin, Jérôme Laurin

**Affiliations:** ^1^Aix Marseille Univ, CNRS, ISM, Marseille, France; ^2^Aix Marseille Univ, INSERM, INMED, Marseille, France; ^3^ONERA, Châtillon, France

**Keywords:** stroke recovery, somatic reflex, thalamus dysfunction, spinal plasticity, mechanical sensibility

## Abstract

The purpose of the present study was to examine the contribution of group III and IV metabosensitive afferents at spinal and supraspinal levels in rats subjected to middle cerebral artery occlusion (MCAO) with reperfusion during the acute phase. Animals were randomized in Control (*n* = 23), SHAM (*n* = 18), MCAO-D1 (*n* = 10), and MCAO-D7 (*n* = 20) groups. Rats performed the Electrical Von Frey and the Adhesive removal tests before the surgery and at day 1 (D1), D3, and D7 after MCAO. Animals were subjected to electrophysiological recordings including the responses of group III/IV metabosensitive afferents to combinations of chemical activators and the *triceps brachii* somatic reflex activity at D1 or D7. The response of ventral posterolateral (VPL) thalamic nuclei was also recorded after group III/IV afferent activation. Histological measurements were performed to assess the infarct size and to confirm the location of the recording electrodes into the VPL. Behavioral results indicated that MCAO induced disorders of both mechanical sensibility and motor coordination of paretic forepaw during 7 days. Moreover, injured animals exhibited an absence of somatic reflex inhibition from the group III/IV afferents at D1, without affecting the response of both these afferents and the VPL. Finally, the regulation of the central motor drive by group III/IV afferents was modified at spinal level during the acute phase of cerebral ischemia and it might contribute to the observed behavioral disturbances.

## Introduction

Hemiparesis constitutes the most common disturbance induced by stroke, which remains the leading cause of motor disability in the world (Lawrence et al., 2001; Hankey et al., 2002). The resulting sensorimotor disorders are frequently associated with an excessive level of neuromuscular fatigue [i.e., reduction in the ability of the muscle to produce force or power, whether the task can be sustained or not, as defined by Barry and Enoka (2007)] when stroke patients performed the least effort. It is considered that such fatigue exacerbates the physical dependence and deconditioning of stroke patients (Colle et al., 2006). Despite these alarming findings, therapeutic advances are hampered by the insufficient knowledge about the stroke-induced pathophysiology of neuromuscular fatigue.

Besides the decrease of cardiovascular fitness and muscular atrophy, the fatigue induced by a sustained contraction is also related to the disturbance of central motor drive, but mechanisms remain poorly understood (Signal et al., 2008; Knorr et al., 2011). It was demonstrated that an exhaustive contraction in both stroke patients and rats with cerebral ischemia revealed a failure in voluntary activation of active muscles (Dormer et al., 2009; Knorr et al., 2012). Moreover, we have previously reported in rats with cerebral ischemia that the greater fatigue of the affected *triceps brachii* during a sustained isometric contraction was parallel with a disinhibition of spinal reflexes (assessed by the Hoffmann reflex or H-reflex) (Pin-Barre et al., 2014). Given that the H-reflex decrease could be partially explained by sensory feedback from active muscle in healthy individuals (Pettorossi et al., 1999; Gandevia, 2001; Laurin et al., 2015), we have postulated that the altered muscle afferents following cerebral ischemia might be involved in the spinal reflexes disinhibition, especially the metabosensitive group III/IV muscle afferents. Indeed, during and after an isometric contraction, the accumulation of metabolites such as potassium, lactic acid (LA), and adenosine triphosphate (ATP), strongly activates molecular receptors located on the terminal end of both thinly myelinated group III and group IV muscle afferent fibers (Kaufman and Rybicki, 1987; Adreani et al., 1997; Amann, 2011). In healthy individuals, their activation could decrease the somatic reflex amplitude of active muscles in order to limit premature peripheral fatigue development (Liu et al., 2002; Klass et al., 2008; Amann et al., 2011; Sidhu et al., 2017). Given that it was not the case following cerebral ischemia, we assessed in the present study the specific role played by these afferents in the perturbation of spinal inhibitory pathways.

In addition, the effects of metabosensitive group III/IV muscle afferents on the central motor drive are not restricted to spinal networks because they also influence the activity of several supraspinal structures (including motor cortex, insular, or cingulate cortex) (Carroll et al., 2017; Sidhu et al., 2017). Some studies suggested that nerve impulses originate from metabosensitive afferents might project to the ventral posterolateral (VPL) nucleus of the thalamus, which is considered as an important relay of somatosensory inputs (Foreman et al., 1979; Kniffki and Mizumura, 1983). It was found that this thalamic structure could be indirectly damaged by cerebral ischemia (Watanabe et al., 1998; Dihné et al., 2002). Therefore, whether the link between group III/IV afferents and VPL activity was confirmed, we postulated that the VPL integration of information from these afferents might be disturbed.

In light of these findings, it thus remains to determine at which levels the effects of metabosensitive afferents on central motor drive might be modulated following cerebral ischemia. The present study was designed to examine the role of metabosensitive group III/IV muscle afferents at the spinal and supraspinal levels in rats with cerebral ischemia. For that, the discharge of metabosensitive afferents to several specific activators was first recorded at the 7th cervical (C7) dorsal root during *in vivo* electrophysiological recordings (Tosolini and Morris, 2012). The injections of activators mimicked the accumulation of metabolites into the muscle observed during and after an exhaustive contraction without activating mechanosensitive afferents. This measurement allowed assessing if cerebral ischemia could induce a desensitization of muscle molecular receptors that could explain a potential change of the somatic reflex response and/or firing rate of VPL. Then, their effects on both somatic reflex amplitude and firing rate of VPL were also highlighted during *in vivo* electrophysiological experiments. All measurements were carried out during the first week following cerebral ischemia where neuroplasticity in the spinal and supraspinal remote areas is the most active and thus constitutes a prime target for treatments (Murphy and Corbett, 2009). We thus hypothesized that the activation of metabosensitive group III/IV muscle afferents might be altered in rats with cerebral ischemia as well as their action on the central motor drive at the spinal and/or supraspinal levels.

## Materials and Methods

### Animals

Overall, 88 adult male Sprague-Dawley rats (250–270 g; JANVIER^®^, France) were used, but 12 of them were excluded. Exclusion criteria come when one of the following points was observed: (1) weight exceeded 270 g before inducing cerebral ischemia, (2) prostrated animals (extreme physical weakness and absence of movement in the cage), (3) more than 20% of their baseline weight lost, and (4) absence of sensorimotor deficits. Based on these exclusion criteria, 12 rats on 42 subjected to middle cerebral artery occlusion (MCAO) were excluded because of death within the first 48 h (*n* = 8), absence of sensorimotor deficits (*n* = 1) and more than 20% of their baseline weight and/or prostrated (*n* = 3).

All rats were housed individually at 23°C under a 12 light/dark cycle. Food and water were provided *ad libitum*. No signs of hyperactivity, anorexia or prostration were observed throughout the protocol duration. Animal’s weight was daily checked. Anesthesia and surgical procedures were performed according to the French Law on Animal Care Guidelines. Animal Care Committees of Aix-Marseille University approved our protocol. Each animal was randomly assigned to one of the five groups:

(1) Control (*n* = 23) for which no surgery for cerebral ischemia was performed. This group enabled to verify the measurements of reliability on 7 days, including electrophysiological recordings that were performed either at the C7 dorsal root of the spinal cord (*n* = 13) or at the (VPL) level (*n* = 10). For the VPL recordings, these animals were first used to confirm that the information from the group III/IV muscle afferents could reach the VPL by increasing its firing rate, and then, they were compared to rats with cerebral ischemia.
(2) SHAM (*n* = 18): animals underwent surgery without cerebral ischemia to ensure that it did not affect measurements and were subjected to electrophysiological recordings either at the C7 dorsal root of the (*n* = 10) or at the thalamic level (*n* = 8).
(3) MCAO-D1 (*n* = 10): animals underwent MCAO and were subjected to electrophysiological recordings at the C7 dorsal root of the spinal cord one day after injury. At day 1 (D1), cerebral edema did not allow acute recordings in the thalamic area.
(4) MCAO-D7 (*n* = 20): animals underwent MCAO and were subjected to electrophysiological recordings 7 days after injury either at the C7 dorsal root of the spinal cord (*n* = 14) or at the thalamic level (*n* = 6).
(5) Pyridoxalphosphate-6-azophenyl-2′,4′-disulfonic acid (PPADS) (*n* = 5): corresponding to additional experiments in which electrophysiological recordings were performed in healthy animals before and after PPADS intra-arterial injection, an antagonist of ATP-gated P2X Receptor Cation Channel (P2X). The aim was to ensure that metabosensitive afferents were mainly stimulated following our chemical injections by blocking one of the most important molecular receptors known to increase the discharge of group III/IV muscle afferents. It might also reveal a potential role of P2X receptors in the somatic reflex regulation.

### MCAO Surgery

Central temperature (maintained at 37–38°C), age, weight, sex, and ischemia duration were rigorously controlled, in order to ensure a reproducible infarct size between animals. Rats were subjected to right MCAO for 2 h. Surgery was described elsewhere (Pin-Barre et al., 2014). Briefly, anesthesia was induced (4%) and maintained (2–2.5%) using isoflurane (Anesteo, Villetelle, France). A 0.2 ml of 0.5% bupivacaine was injected subcutaneously along the prospective incision site 30 min before surgery. The right external, internal, and common carotid arteries (ECA, ICA, and CCA) were exposed. After a partial arteriotomy on ECA, a 0–4 monofilament nylon suture (silicon-coated tip length and diameter: 5 mm and 0.39 ± 0.02 mm, respectively; Redland, CA, United States) was inserted into the ICA via the ECA and approximately pushed 20 mm away from the carotid bifurcation. Blood flow was thus blocked at the middle cerebral artery (MCA) origin during 120 min. Then, the monofilament was removed to allow reperfusion and skin was sutured. Finally, an administration of buprenorphine (0.05 mg/kg) into the peritoneal space was performed before replacing the animal in its box (Percie du Sert et al., 2017).

### Behavioral Tests

**FIGURE 1:**
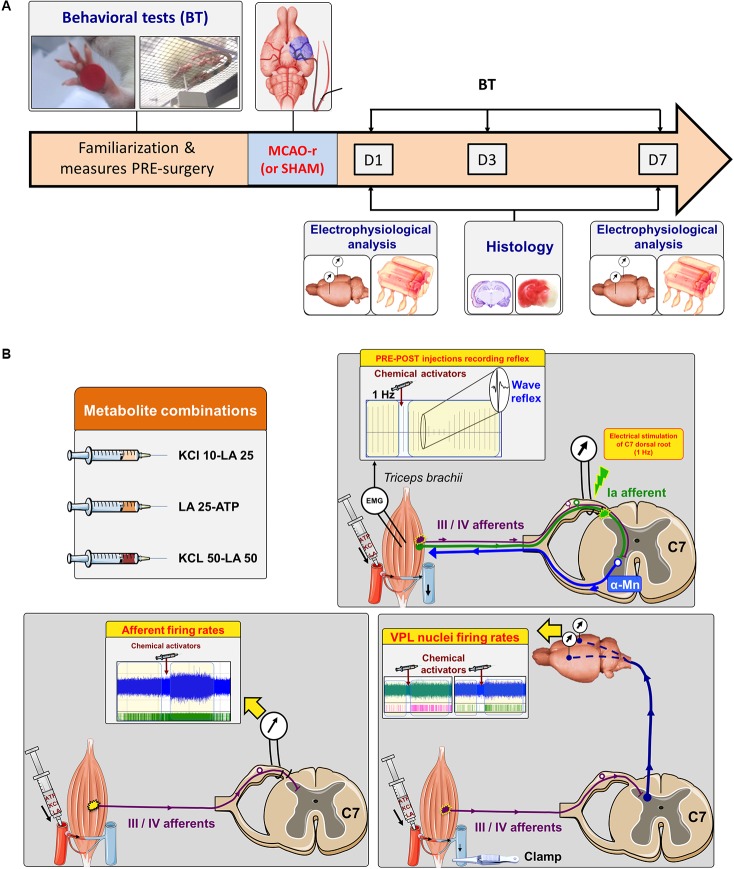
Experimental protocol during 7 days post-MCAO and electrophysiological procedures. **(A)** Electrophysiological recordings at the spinal cord or VPL levels were performed at day 1 (D1) or day 7 (D7) post-MCAO before histological analysis. Moreover, animals sacrificed at D7 also carried out the behavioral tests at D1, D3, and D7. **(B)** Experimental procedures for the somatic reflex recording evoked at the C7 dorsal root (top right), the recording of afferent firing rate at the C7 dorsal root (bottom left) and for the recording of both VPL nuclei firing rate (bottom right). α-Mn: α-motoneurons; C7: cervical 7.

To determine the influence of cerebral ischemia on sensorimotor deficits, animals were subjected to Electronic Von Frey (EVF) test and Adhesive removal test (ART) before (PRE) and at 1, 3, and 7 days (D1, D3, and D7, respectively) after MCAO surgery (**Figure 1A**). Rats were familiarized with these tests four times per week during 2 weeks to reduce stress and inter-/intra-individual variability. Moreover, the same experimenter carried out measurements in blind.

#### Electronic Von Frey (EVF) Test

Mechanical sensibility threshold to pressure of both forepaws and hindpaws was assessed using an EVF device (EVF, Bioseb Apparatus, Vitrolles, France) (Martinov et al., 2013). First, rat was placed individually on mesh floor stand, located at height, before covering animal with a plastic box to avoid mobility outside of the mesh. Acclimation to the environment lasted 15 min prior each measurement and the recording started once animal was quiet and in static position. Then, a mechanical pressure was progressively applied to each paw with a plastic tip, positioned to a probe connected to EVF unit. The plastic tip reached the smooth part of the paw by passing through the opening of the mesh floor. Maximal force (in grams) was then recorded when the animal induced withdrawal of the paw. Each paw underwent six trials separated by 1 min to avoid desensitization. If the animal did not remove its paw after 100 g, the trial was stopped and 100 g was the recorded value. Minimal and maximal values were excluded and the four trials remaining were averaged. Ratios of both left forepaw/right forepaws (LF/RF) and left hindpaw/right hindpaws (LH/RH) were calculated.

#### Adhesive Removal Test (ART)

Adhesive removal test (ART) was used to independently assess tactile sensibility and motor coordination of both left and right forepaws (ART-LF and ART-RF, respectively) (Schallert et al., 1982; Schallert, 2006). Animal was placed within a plastic box (45 cm × 60 cm) and was acclimated to the environment during 3 min prior measurements. Then, a round sticky label (8 mm) was randomly attached to smooth part of the left or right forepaw. The time to contact (TC) was recorded (in s), informing about tactile sensory deficits, as well as the time to remove (TR) the sticky label, focusing on motor coordination deficits. Two trials were performed for each paw to prevent sensitization.

### Electrophysiological Analysis

#### Animal Preparation

Rats were anesthetized with an intra-peritoneal injection of urethane solution (100 mg/100 g). Central temperature was maintained at 37°C driven by a heater blanket (Harvard Homeothermic Blanket Control Unit, K01345CE). Electrophysiological recordings were acquired either at C7 dorsal root level of the spinal cord or at thalamic level in both ipsi- and contralesional (IP and CT) VPL nuclei. For each procedure, the first step of surgery was common and involved inserting a catheter in a collateral artery of the brachial artery in order to inject group III and IV chemical activators in the blood toward the *triceps brachii*. Animals were placed in dorsal *decubitus* and the *pectoralis major* and *minor* muscles were removed to expose the *musculospiral* nerve, the *brachial* artery and the *brachial* vein (Angélica-Almeida et al., 2013). All nerves surrounding the *musculospiral* nerve were sectioned to avoid any influences during electrophysiological recordings. The *musculospiral* nerve was kept intact throughout surgeries. Then, a hemisection was performed in the brachial artery collateral in which a catheter was inserted in order to inject activators of group III/IV afferents toward the *triceps brachii*. Thus, the *brachial* artery was kept intact during such procedure, preserving blood flow to the *triceps brachii*. After this step, animals were placed in ventral *decubitus* position for either reflex or thalamic recordings.

To record both the amplitude of the somatic reflex and the firing rate of group III/IV muscle afferents, the C7 dorsal root ganglion (DRG), including *triceps brachii* afferent fibers (Tosolini and Morris, 2012), was exposed and was kept intact. For that, a laminectomy was performed from C5 to T1 vertebrae. Then, the *dura* was removed from C6 to C8 and dorsal roots were immersed in paraffin oil. Finally, the left forepaw was strongly fixed at 130°.

#### Electrically Evoked Somatic Reflex

Single rectangular shocks were directly applied to the intact C7 dorsal root using a pair of tungsten hook electrodes with a 1-ms pulse generated by a constant current stimulator (Digitimer DS7 A; Hertforshire, United Kingdom) (**Figure 1B**). The somatic reflex was recorded using bipolar needle electrodes (29-gauge; AD Instruments Ltd., Oxford, United Kingdom; MLA 1204 needle electrodes, 2-mm pin) inserted into the belly of the *triceps brachii* muscle. The location of the electrodes was never changed throughout recordings (between PRE- and POST-injection measurements). The reflex signal was referred to a ground electrode implanted in an inert tissue, amplified (2k), and filtered (30 Hz to 10 kHz) with a differential amplifier (P2MP^®^, 5104B, Marseille, France) (Laurin et al., 2016). The maximal peak-to-peak amplitude of the somatic reflex wave was determined by incrementally increasing stimulation intensity (by 0.01-mA increments) from 0.05 mA until there was no further wave amplitude increment. Once the maximal somatic reflex wave was determined, a series of 20 reflexes were evoked at 1 Hz during 20 s to control reflex amplitude stability. After 15 min of rest to reduce muscular fatigue, the reflex amplitude was recorded before and after the intra-arterial injection (0.4 ml) of the 3 following combinations of metabosensitive afferent activators at doses fixed in accordance with previous works (Thimm and Baum, 1987; Hanna and Kaufman, 2003; Light et al., 2008; Laurin et al., 2016): 10 mM of potassium chloride (KCl) and 25 mM LA (KCl 10-LA 25), 25 mM of LA and 50 μg/kg of adenosine triphosphate (LA 25-ATP), and 50 mM of KCl and 50 mM of LA (KCl 50-LA 50). The injection of these three combinations was randomized across animals to avoid habituation/sensitization effects. Specifically, 6 reflexes were evoked before (PRE-injection) and a series of 20 reflexes were elicited after the injections (POST-injection) (Laurin et al., 2010, 2016). Each of the 3 combinations was delivered every 12 min to ensure that muscular environment was returned to baseline condition (Rybicki et al., 1985). This procedure allowed assessing the impact of metabosensitive afferents on somatic reflex. The first 6 reflexes were averaged. Concerning the 20 POST-injection reflexes, 2 successive amplitudes of reflexes were averaged until the end of recording (the 1st with the 2nd reflex, then the 2nd with 3rd, then 3rd with the 4th…) and compared to the 6 averaged reference reflexes in order to detect the maximal variation of reflex amplitude. More precisely, the effect of afferent activation on reflex could be observed on a few evoked reflexes. We did not average the 20 reflexes because the variation of amplitude should be hidden. The maximal peak variation of somatic reflex amplitude was expressed in the percentage (%) of PRE. The Reflex-POST/Reflex-PRE injection ratio was calculated. In addition, reflexes were also elicited before and after saline injection in order to confirm that only specific chemical activators of metabosensitive afferents influenced the reflex responses. Some methodological points indicated that recording conditions of somatic reflex at the dorsal root were similar for each animal: (1) somatic reflex wave gradually increased with stimulation intensity until reaching the maximal wave and (2) the latency of reflex wave was similar from one animal to another.

#### Firing Rate Recording of Metabosensitive Muscle Afferents

The recording of afferent discharge was performed at the dorsal root. It allowed us to acutely detect sensory signal than on entire nerve, which include both sensory and motor fibers. A pair of bipolar tungsten electrodes was thus positioned on C7 dorsal root before being immersed in paraffin oil. Electrode position on dorsal root was never changed throughout recordings. The neural signal was amplified (10–100 K) and filtered (30–10 kHz) with a differential amplifier and was also referred to a ground electrode implanted in a nearby tissue. The nervous activity was recorded and fed into pulse window discriminators (P2MP^®^; SARL), from which the firing rate was extracted. The output of these discriminators providing noise-free tracings (discriminated units) was displayed on a computer on separate tracings using a data acquisition system (Biopac MP150^®^ and AcqKnowledge software, Paris, France). Before injecting the chemical activators, a baseline recording was performed to ensure that the afferent firing rate was stable. For that, the impulse activity was recorded during 210 s without chemical injection. The recording was considered acceptable if the fluctuation of baseline impulse activity did not exceed ± 10% throughout the recording. Once the baseline was established, firing rate of metabosensitive afferents were recorded before (60 s; PRE) and after (150 s; POST-injection) the intra-arterial injection of the above three activator combinations (**Figure 1B**). Baseline firing rate recording could also be repeated between chemical injections. The 3 injections were separated by 12 min of rest (Rybicki et al., 1985). Prior each injection, brachial vein of the *triceps brachii* was clamped in order to block venous return and avoid a reflex wave influence from the diffusion of chemical activators in the blood. Consequently, the recorded reflex amplitude was related to the activation of metabosensitive muscle afferents (**Figure 1B**). The post-injection firing rate was compared with the corresponding baseline. The temporal profile of firing rate variation and the extraction of the peak firing rate after each injection were performed by a specific MATLAB program (The MathWorks, version 8.0.0.783 R2012b) that was described elsewhere (Laurin et al., 2011). Briefly, the firing rate was measured on 10 s periods all along the recording period, in which the firing rate was calculated on different time interval lasting from 1 to 10 s and averaged between them. The variations were expressed in percentage of the corresponding baseline discharge (i.e., baseline firing correspond to 0%) as well as in the AFF-POST/AFF-PRE injection ratio.

#### Firing Rate of VPL Nuclei

The rat head was strongly fixed in a stereotaxic device (WPI^®^, World Precision Instruments, Hertfordshire, United Kingdom). The *bregma* was exposed. VPL nuclei coordinates were determined according to Paxinos and Watson (2006): 2.9 mm for dorsoventral axis, -6.3 mm for mediolateral axis, and -2.28 mm for the rostrocaudal axis. A craniotomy (4 mm^2^) was then performed at the predefined coordinate points using a power drill (WPI^®^, World Precision Instruments, Hertfordshire, United Kingdom). The *dura* was removed. This procedure was repeated for each hemisphere. Then, a pair of platinum bipolar electrodes was inserted into the IP- and CT VPL nuclei. Prior each injection, brachial vein of the *triceps brachii* was clamped in order to block venous return and avoid the VPL nuclei activation via the diffusion of chemical activators in the blood. Consequently, the recorded firing rate in these areas was related to the activation of metabosensitive muscle afferents (**Figure 1B**). The recording protocol and the MATLAB treatment were the same as for metabosensitive afferents recording described above. The firing rate variation after injection was first compared with the corresponding baseline discharge. Then, these values were expressed in percentage to the variation of the firing rate following saline injection in each hemisphere (IP-PRE and CT-PRE). Ratios of IP-POST/IP-PRE and CT-POST/CT-PRE were calculated.

#### P2X Receptor Blockade With PPADS

To confirm that KCl, LA, and ATP combinations specifically activated the molecular P2X receptors of group III/IV muscle afferents, both somatic reflex amplitude and afferent firing rate were recorded before and 30 min after an intra-arterial PPADS injection into the *triceps brachii*. PPADS is an antagonist of ATP-gated P2X receptors. PPADS agent was dissolved in saline (10 mg/kg, 0.2 ml) as previously described (Hanna and Kaufman, 2003; Kindig et al., 2007; Wang et al., 2010). Prior to the injection, the collateral of axillary artery and the venous return were clamped during 15 min for blocking PPADS in the blood vessels of the *triceps brachii*. Then, clamps were removed to allow muscle reperfusion. The orange color of PPADS allowed us to control if *triceps brachii* was well supplied by this agent. Otherwise, recordings were not retained. Only KCl 10-LA 25 and LA 25 –ATP injections were administered to limit the amount of substances for the same animal.

### Immunohistochemistry Analysis

Once the electrophysiological analysis completed, animal remained under anesthesia and was assigned either to transcardiac perfusion or decapitation to perform cresyl violet staining (to control electrode location within VPL area) or 2,3,5-triphenyl-2H-tetrazolium chloride (TTC) staining to assess infarct size at D1 or D7.

#### Cresyl Violet Staining

Transcardiac perfusion was performed (25 ml/min) with 250–300 ml of cold phosphate-buffer saline (PBS-1X, Sigma-Aldrich^®^) followed by the same amount of paraformaldehyde 3% (Antigenfix, Sigma-Aldrich^®^). Then, brain tissues were removed and successively post-fixed in Antigenfix at 4°C during 24 h and cryoprotected in 30% of sucrose. Finally, tissues were flash-frozen in isopentane and stored at -80°C. Coronal sections (30 μm) of rat brain area surrounding electrode insertion point were performed before mounting onto Superfrost Plus glass slides. Sections were first rinsed in distilled water for 5 min, incubated 3 min in a cresyl violet bath and then dehydrated through a sequence of ethanol baths (70°, 95°, and 100°). They were finally cleaned in Xylene solution during 2 min and mounted using Permount (Fair Lawn, NJ, United States). Image acquisitions were performed with a BX50 Olympus microscope equipped with a digital camera and X4 optical lens. We controlled the coordinates of the electrode insertion in each hemisphere according to Rat brain Atlas of thalamic VPL (*bregma*: -2.28 mm) (Paxinos and Watson, 2006).

#### TTC Staining

Anesthetized animals were decapitated. Fresh brain tissues were quickly removed and placed at -80°C during 4 min before performing a 2-mm-thick coronal sections using a brain slicer device. Coronal sections were then dropped in 5 ml of 1% TTC solution (Sigma-Aldrich^®^) during 30 min at 37°C and washed 3 times with PBS-1X and fixed with Antigenfix during 24 h. Infarcted tissues (white-colored area) were delimited with ImageJ software and infarct size was expressed in percentage of both right hemisphere and total brain volume.

### Statistical Analysis

Statistical analysis was performed using SigmaStat^®^ software program (San Jose, CA, United States). All data are presented as Mean ± SD. Raw values of behavioral tests from D1 to D7 were averaged and compared to PRE value between groups with a two-way ANOVA (time × groups). For electrophysiological analysis, ratios of AFF-POST/AFF-PRE, Reflex-POST/Reflex-PRE, the IP-POST/IP-PRE, and CT-POST/CT-PRE were averaged in each group and compared between groups with a one-way ANOVA. *Post hoc* comparisons were performed with Student–Newman–Keuls multiple post-test comparisons. To compute the magnitude of difference between groups, the effect size was calculated for main results using Cohen’s *d* for significant differences. Large effect size was considered for Cohen’s *d* ≥ 0.9, medium for Cohen’s *d* between 0.9 and 0.5 and small for Cohen’s *d* between 0.5 and 0.25. Moreover, in each group, raw values of PRE and POST injections were average and compared with Student’s *t*-test for group III/IV afferents and VPL firing rates as well as for somatic reflex amplitudes. Likewise, the PRE-PPADS and POST-PPADS injections for group III/IV afferents firing rate as well as somatic reflex amplitudes were also compared with Student’s *t*-test. Results were significant when *p* < 0.05.

## Results

### Behavioral Tests

For both EVF and ARTs, no difference was observed in Control and SHAM groups from D1 to D7 compared to PRE. Furthermore, PRE values were not different between Control, SHAM, and MCAO-D7 groups.

#### Electronic Von Frey (EVF) Test

The mechanical sensibility of the paretic forepaw decreased in the acute phase of cerebral ischemia (**Table 1**). Indeed, for MCAO-D7 group, the mechanical sensibility threshold to pressure of the LF (paretic side) was significantly increased at D1, D3, and D7 compared to PRE (*p* < 0.001). This threshold was also higher to the one of the Control and SHAM groups from D1 to D7 (*p* < 0.001).

**Table 1:** Behavioral tests for Control, SHAM, and MCAO-D7 groups.

		Control				SHAM				MCAO-D7			
		PRE	D1	D3	D7	PRE	D1	D3	D7	PRE	D1	D3	D7
Electrical Von Frey	LF (g)	32.6 ± 8.4	31.4 ± 8.8	26.7 ± 3.8	23.7 ± 6.7	34.2 ± 10.0	34.2 ± 9.4	39.4 ± 13.9	31.5 ± 8	30.6 ± 8.6	64.3 ± 18.8**^∗/+^**	68.8 ± 11.3**^∗/+^**	76.2 ± 9.2**^∗/+^**
	LF/RF	1.1 ± 0.3	1.1 ± 0.2	1.1 ± 0.4	1.0 ± 0.2	1.1 ± 0.2	1.1 ± 0.1	1.2 ± 0.2	1.0 ± 0.2	1.1 ± 0.3	2.6 ± 0.8**^∗/+^**	2.4 ± 0.9**^∗/+^**	2.0 ± 0.5**^∗/+/Φ^**
Adhesive removal test (in s)	TD-LF	3.2 ± 2.6	4.5 ± 4.0	1.9 ± 1.4	3.1 ± 3.0	5.1 ± 9.2	2.8 ± 2.9	4.4 ± 3.0	2.0 ± 1.4	5.6 ± 5.4	141.4 ± 115.1**^∗/+^**	95.1 ± 100.7**^∗/+/Φ^**	85.4 ± 65.4**^∗/+/Φ^**
	TR-LF	14.8 ± 8.3	16.3 ± 12.6	7.9 ± 6.5	11.9 ± 13.4	13.6 ± 13.7	13.9 ± 15.5	8.8 ± 4.1	8.8 ± 5.8	11.7 ± 7.1	279.0 ± 32.7**^∗/+^**	217.3 ± 107.0**^∗/+/Φ^**	173.0 ± 91.1**^∗/+/Φ/#^**
	TD-RF	4.9 ± 7.8	3.6 ± 4.1	4.7 ± 4.9	1.5 ± 1.2	5.0 ± 3.7	3.8 ± 3.4	3.6 ± 4.1	3.3 ± 3.1	7.3 ± 5.7	12.4 ± 15.5**^+^**	6.9 ± 7.1**^+^**	3.8 ± 4.1**^+/Φ^**
	TR-RF	8.4 ± 9.5	8.9 ± 6.8	9.3 ± 11.3	7.5 ± 8.7	12.3 ± 9.4	11.4 ± 8.2	6.5 ± 4.3	11.6 ± 8.6	15.8 ± 8.9	73.6 ± 61.3**^∗/+^**	69.6 ± 105.0**^∗/+^**	22.4 ± 24.1**^Φ^**

*Data represent Mean ± SD. ^∗^ indicates significant difference between PRE and D1 to D7 in MCAO-D7 group; + indicates significant difference between MCAO-D7 group and Control and SHAM groups; Φ indicates significant difference between D1 and D3 to D7 in MCAO-D7 group; # indicates significant difference between D7 and D3 in MCAO-D7 group (p < 0.05; see the section “Results” for details for p-values). LF: left forepaw; LF/RF: left forepaw/right forepaw ratio; RF: right forepaw; TD: time to detect; TR: time to remove.*

However, results showed that the mechanical sensibility threshold to pressure of both the RF and the hindpaws were not changed from D1 to D7 compared to PRE in MCAO-D7 group. Likewise, no difference was observed between groups from D1 to D7 (data not shown).

The LF/RF ratio of the mechanical sensibility threshold was significantly increased at D1, D3, and D7 compared to PRE for MCAO-D7 group (*p* < 0.001). However, this ratio was significantly decreased at D7 compared to D1 (*p* < 0.01) and D3 (*p* < 0.05). Moreover, LF/RF ratio was significantly higher from D1 to D7 for MCAO-D7 group compared to Control and SHAM groups (*p* < 0.001).

#### Adhesive Removal Test (ART)

The tactile sensibility and the motor coordination were affected in the acute phase of cerebral ischemia both for paretic and for non-paretic forepaws (**Table 1**).

##### Left forepaw

The TD-LF was significantly increased at D1 (*p* < 0.001), D3 (*p* < 0.05), and D7 (*p* < 0.05) compared to PRE for MCAO-D7 group. However, a significant decrease of the TD-LF was observed for MCAO-D7 at D3 and D7 compared to D1 (*p* < 0.01). Furthermore, the TD-LF was significantly higher in the MCAO-D7 group compared to Control and SHAM groups at D1 (*p* < 0.001), D3 (*p* < 0.05 and *p* < 0.01, respectively) and at D7 (*p* < 0.05).

The TR-LF was significantly increased in the MCAO-D7 at D1, D3, and D7 compared to PRE (*p* < 0.001). However, a significant decrease of the TR-LF was observed for MCAO-D7 at D3 and D7 compared to D1 (*p* < 0.001), and also at D7 compared to D3 (*p* < 0.001). Furthermore, the TR was significantly higher in the MCAO-D7 compared to Control and SHAM groups from D1 to D7 (*p* < 0.001).

##### Right forepaw

The TD-RF was significantly decreased at D7 compared to D1 in the MCAO-D7 group (*p* < 0.05). This time was significantly higher in the MCAO-D7 group compared to Control and SHAM groups at D1 (*p* < 0.001), D3 (*p* < 0.05 and *p* < 0.001, respectively) and at D7 (*p* < 0.05).

The TR-RF was significantly increased in the MCAO-D7 at D1 and D3 compared to PRE (*p* < 0.01). However, the TR-RF was significantly decreased at D7 compared to D1 and D3 in the MCAO-D7 group (*p* < 0.01). The TR was significantly higher in the MCAO-D7 compared to Control and SHAM groups at D1 and D3 (*p* < 0.001).

### Electrophysiological Analysis

#### Response of Somatic Reflex After Injection of Group III/IV Chemical Activators

**FIGURE 2:**
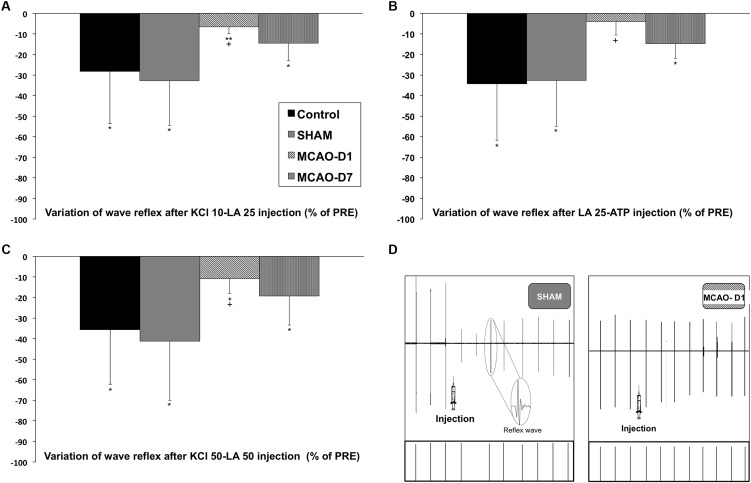
Changes in somatic reflex response following **(A)** KCl 10-LA 25, **(B)** LA 25-ATP, and **(C)** KCl 50-LA 50 injections in the *triceps brachii*. Values are expressed in the percentage (%) of PRE amplitude reflex. ^∗^*p* < 0.05, ^∗∗^*p* < 0.01: PRE-injections vs. POST-injections in Control, SHAM, MCAO-D1, and MCAO-D7 groups; + *p* < 0.05: MCAO-D1 vs. Control and SHAM groups. **(D)** Examples of somatic reflex responses to metabosensitive afferent stimulations between SHAM and MCAO-D1 groups. The amplitude of the somatic reflex was not modified following chemical activator injections for MCAO-D1 animal unlike the one of the SHAM animal.

The somatic reflex amplitude of the *triceps brachii* was not decreased by group III/IV muscle afferents following cerebral ischemia. Results showed that following KCl 10-LA 25 injection the amplitude reflex was significantly decreased compared to PRE-injection for Control (-28 ± 25.6%; *p* < 0.05), SHAM (-32.8 ± 21.7%; *p* < 0.05), MCAO-D1 (-6.6 ± 3.3%; *p* < 0.01), and MCAO-D7 (-14.6 ± 8.5%; *p* < 0.05) (**Figure 2A**). However, the depression of reflex amplitude was significantly lower in MCAO-D1 group than in Control and SHAM (*p* < 0.05; Cohen’s *d* = 0.4).

Likewise, we observed similar results after LA 25-ATP injection for Control, SHAM, and MCAO-D7 groups (-34.2 ± 27.5; -32.6 ± 22.3; -14.8 ± 7.1%, respectively; *p* < 0.05 for all) (**Figure 2B**) as well as after KCl 50-LA 50 injection for Control, SHAM, and MCAO-D7 (-35.5 ± 26.7; -41.2 ± 28.9; -19.2 ± 14.2%, respectively; *p* < 0.05 for all) (**Figure 2C**). However, the reflex amplitude after LA 25-ATP (-4.0% ± 6.5%) and KCl 50-LA 50 (-10.9% ± 7.1%) injections was not significantly reduced in MCAO-D1 compared to PRE-injection. Moreover, the variation of reflex amplitude was significantly lower in MCAO-D1 group than in Control and SHAM groups after LA 25-ATP and 50-LA 50 injections (*p* < 0.05 for all; Cohen’s *d* = 0.6 and *d* = 0.4, respectively). Finally, no difference was found between MCAO-D1 and MCAO-D7 groups for all chemical activator combinations. An example of reflex measurement before and after injection was given in **Figure 2D**.

#### Response of Group III/IV Afferents After Chemical Injections

**FIGURE 3:**
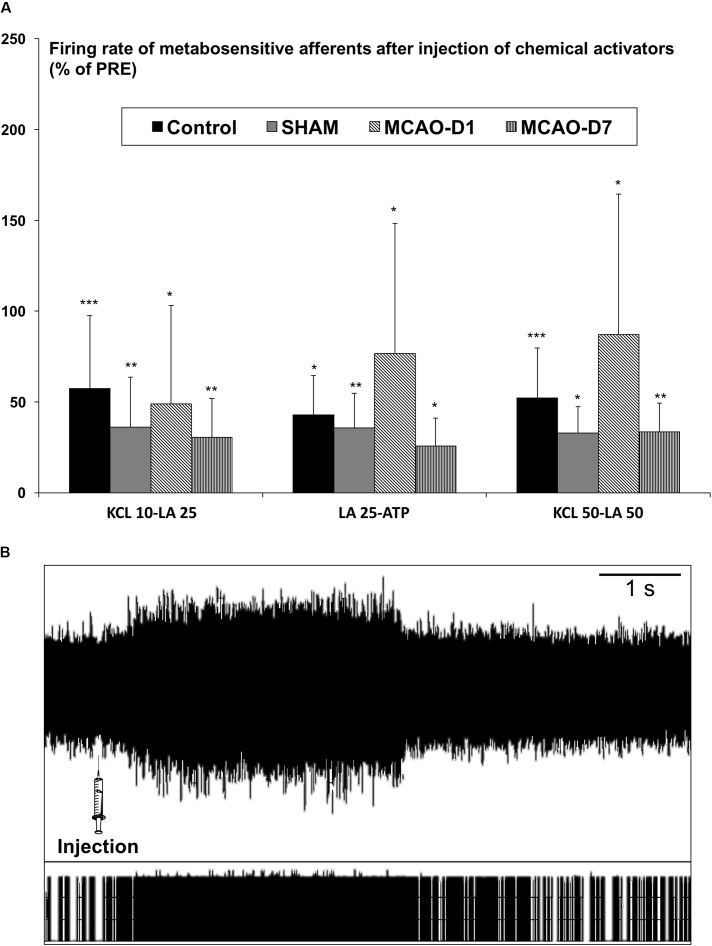
Changes in the discharge of metabosensitive afferent after intra-arterial injection of chemical activators in the *triceps brachii*. **(A)** Values are expressed in the percentage (%) of firing rate baseline (PRE). ^∗^*p* < 0.05, ^∗∗^*p* < 0.01, ^∗∗∗^*p* < 0.001: POST-injections vs. PRE-injections in Control (*n* = 13), SHAM (*n* = 10), MCAO-D1 (*n* = 10), and MCAO-D7 (*n* = 14) groups. **(B)** Examples of metabosensitive afferents firing rate after KCl 50-LA 50 in rat of Control group.

The discharge of metabosensitive muscle afferents was not different between non-injured and injured animals. Indeed, the firing rate of metabosensitive afferents was significantly increased compared to PRE-injection for all groups following KCl 10-LA 25 (Control: 57.4 ± 40.1%, *p* < 0.001; SHAM: 36.2 ± 27.5%, *p* < 0.01; MCAO-D1: 49 ± 54%, *p* < 0.05; MCAO-D7: 30.5 ± 21.5%, *p* < 0.01), LA 25-ATP (Control: 42.8 ± 21.8%, *p* < 0.05; SHAM: 35.6 ± 19.2%, *p* < 0.01; MCAO-D1: 76.7 ± 71.6%, *p* < 0.05; MCAO-D7: 25.7 ± 15.4%, *p* < 0.05), and KCl 50-LA 50 injections (Control: 52.2 ± 27.5%, *p* < 0.001; SHAM: 32.8 ± 14.6%, *p* < 0.05; MCAO-D1: 87.1 ± 77.5%, *p* < 0.05; MCAO-D7: 33.5 ± 15.8%, *p* < 0.01) (**Figure 3A**). An example of discharge before and after injection was given in **Figure 3B**. However, the AFF-POST/AFF-PRE ratio was not significantly different between Control, SHAM, MCAO-D1, and MCAO-D7 groups after the various combinations of chemical activators (data not shown).

#### Response of VPL Nuclei After Chemical Injections

**FIGURE 4:**
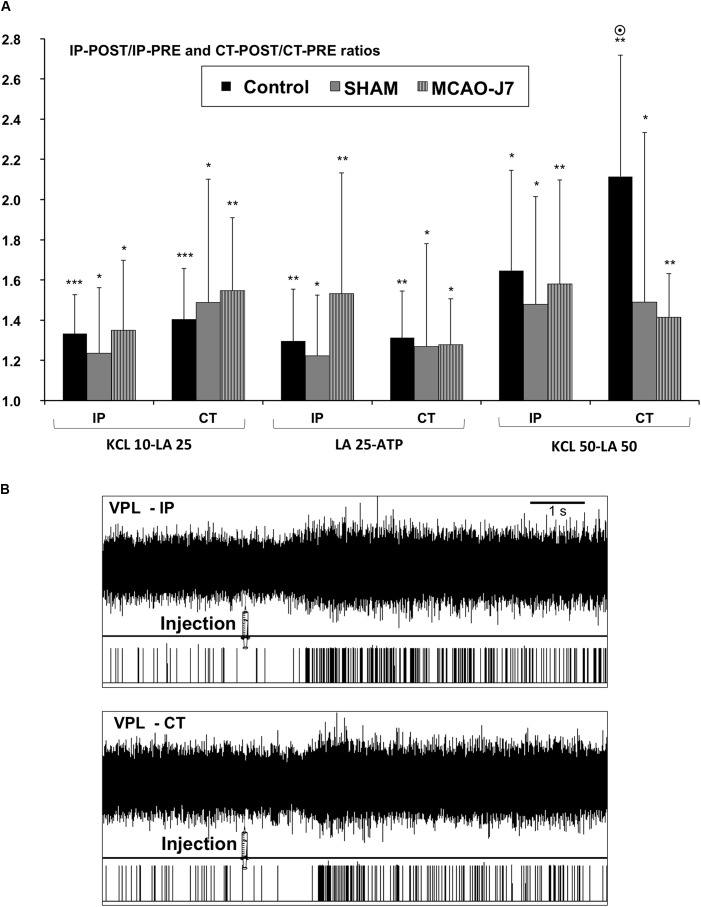
Changes in ipsi- (IP) and contralesional (CT) VPL nuclei response following intra-arterial injection of chemical activators in the *triceps brachii*. **(A)** Values are expressed in IP-POST/IP-PRE and CT-POST/CT-PRE ratios in which PRE corresponded to VPL nuclei baseline in responses to saline injection. ^∗^*p* < 0.05, ^∗∗^*p* < 0.01, ^∗∗∗^*p* < 0.001: POST-injections vs. PRE-injections in Control (*n* = 10), SHAM (*n* = 8), and MCAO-D7 (*n* = 6) groups. ^⊙^*p* < 0.05: IP-POST/IP-PRE vs. CT-POST/CT-PRE in Control group after KCl 50-LA 50 injections only. **(B)** Example of IP and CT VPL nuclei firing rate after KCl 50-LA 50 from a Control rat. IP: ipsilesional; CT: contralesional.

The discharge of VPL nuclei following the activation of group III/IV muscle afferents was not affected by cerebral ischemia. Indeed, the firing rate of IP VPL significantly increased compared to PHY-injection for all groups (**Figure 4A**) following KCl 10-LA 25 (Control: 40 ± 14%, *p* < 0.001; SHAM: 40 ± 28%, *p* < 0.05; MCAO-D7: 45 ± 28%, *p* < 0.05), LA 25-ATP (Control: 38 ± 24%, *p* < 0.01; SHAM: 49 ± 13%, *p* < 0.05; MCAO-D7: 53 ± 60%, *p* < 0.01), and KCl 50-LA 50 injections (Control: 73 ± 47%, *p* < 0.05; SHAM: 79 ± 43%, *p* < 0.05; MCAO-D7: 89 ± 25%, *p* < 0.01). Similar results were found for CT VPL nucleus after KCl 10-LA 25 (Control: 44 ± 24%, *p* < 0.001; SHAM: 67 ± 41%, *p* < 0.05; MCAO-D7: 55 ± 36%, *p* < 0.01), LA 25-ATP (Control: 41 ± 17%, *p* < 0.01; SHAM: 45 ± 29%, *p* < 0.05; MCAO-D7: 36 ± 14%, *p* < 0.05), and KCl 50-LA 50 injections (Control: 98 ± 52%, *p* < 0.01; SHAM: 46 ± 36%, *p* < 0.05; MCAO-D7: 41 ± 22%, *p* < 0.01). Nevertheless, we found no difference between groups both for the IP-POST/IP-PRE and CT-POST/CT-PRE ratios (data not shown). Moreover, we compared the IP-POST/IP-PRE ratio with CT-POST/CT-PRE ratio and no difference was observed in SHAM and MCAO-D7 groups following the 3 combinations of chemical activators as well as in Control group for KCl 10-LA 25 and LA 25-ATP (data not shown). We only observed a significant lower IP-POST/IP-PRE ratio (1.65 ± 0.50) compared to CT-POST/CT-PRE ratio (2.11 ± 0.60) within the Control group in response to KCl 50-LA 50 (*p* < 0.05). An example of VPL firing rate before and after injection was given in **Figure 4B**.

#### Effect of PPADS on the Somatic Reflex Response and on the Discharge of Group III/IV Afferents in Response to Chemical Activators

**FIGURE 5:**
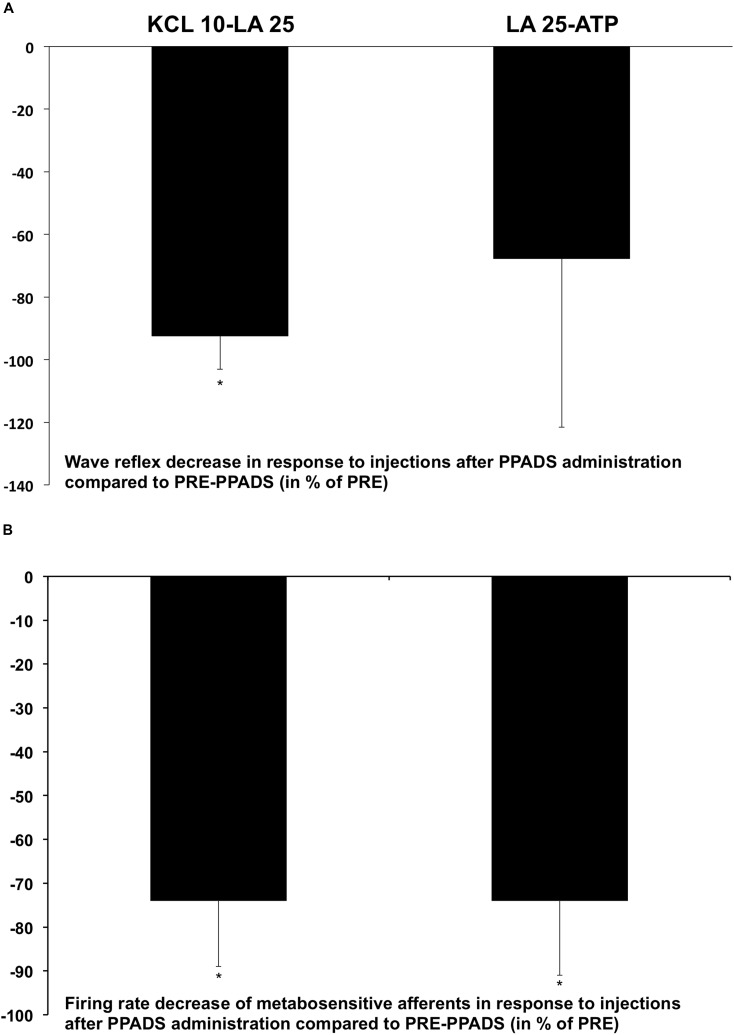
PPADS injection reduced **(A)** somatic reflex and **(B)** metabosensitive afferent responses following chemical activator injections. Values are expressed in the percentage (%) of decrease compared to PRE-PPADS variation in additional animals (*n* = 5). ^∗^*p* < 0.05: POST-PPADS vs. PRE-PPADS.

The PPADS reduced the metabosensitive afferent discharge and the somatic reflex response to chemical activators. Our results showed that the firing rate of metabosensitive afferents was significantly lower in POST-PPADS than in PRE-PPADS injection in response to KCl 10-LA 25 (-74 ± 15%; *p* < 0.05) and LA 25-ATP (-74 ± 17%; *p* < 0.05) injections (**Figure 5A**). Similarly, somatic reflex amplitude was significantly reduced at POST-PPADS compared to PRE-PPADS in response to KCl 10-LA 25 (-92 ± 11%; *p* < 0.05) whereas reduction is found after LA 25-ATP injections (-68 ± 54%) but it was not significant (**Figure 5B**).

### Immunohistochemistry

#### Cresyl Violet Staining

**FIGURE 6:**
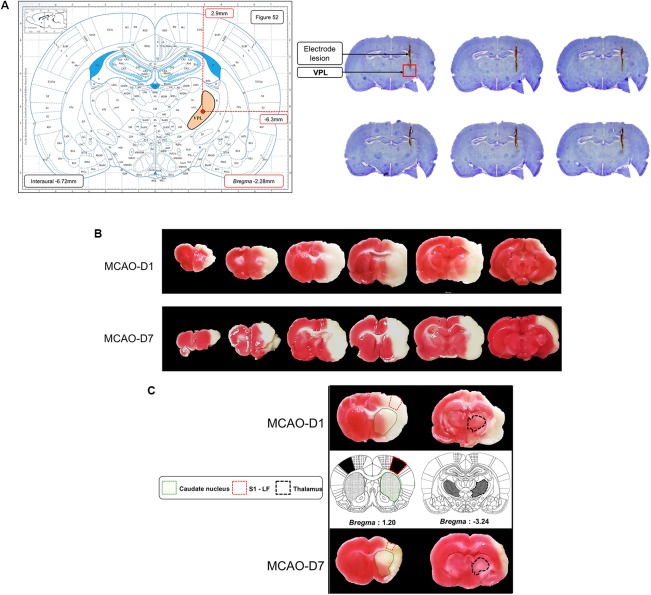
Histological analysis. **(A)** Cresyl violet staining (top right picture) revealed that electrodes were located into VPL nucleus according to the Figure 52 of Paxinos and Watson (2006) (top left picture). In this schematic representation of coronal brain slice, VPL nucleus area was colored in orange. **(B)** TTC staining revealed infarct size (white-colored area) in whole brain slices at D1 and D7 after 2 h of MCAO surgery. **(C)** Affected brain regions by MCAO-induced ischemia. In our study, caudate nucleus (striatum) and primary somatosensory area of left forepaw (S1-LF) were affected by cerebral ischemia at D1 as well as at D7 according to Paxinos and Watson (2006) brain atlas. Nevertheless, the thalamus seemed to be intact at macroscopic level.

We confirmed that the electrode were located into the VPL nuclei with cresyl violet staining based on the Figure 52 of Paxinos and Watson (2006) as illustrated on **Figure 6A**.

#### TTC Staining

To control the reproducibility of infarct size, brain tissues were sectioned and stained with TTC. Our results showed that infarct size represented 29 ± 7% of total brain and 56 ± 12% of right hemisphere at D1. At D7, infarct size was not different than the one at D1 (**Figure 6B**). Moreover, we observed from a quality standpoint that brain ischemia affected the somatosensorial cortex and a part of the striatum. We also noticed that cerebral ischemia did not affect thalamic area at both D1 and D7 (**Figure 6C**).

## Discussion

We first provided evidence that the regulation of spinal somatic reflex by metabosensitive muscle afferents was affected at the spinal level during the acute phase of cerebral ischemia. This finding seemed not to be associated with a change of the metaboreceptor response to stimuli. Moreover, cerebral ischemia did not affect the VPL activity following group III and IV afferent activation. Nevertheless, we also demonstrated in healthy animals that VPL nuclei seemed to receive the information arising from group III and IV metabosensitive afferents and that P2X receptors might be involved in the somatic reflex regulation.

### Methodological Considerations and Limitations

The MCAO surgery by itself had no influence on the measured parameters as indicated by SHAM results. We could thus assume that our results were related to cerebral ischemia. In addition, TTC staining of brain damages and the strong sensorimotor disorders confirmed that the lesion severity seemed to be reproducible between animals 24 h after MCAO.

Combinations of group III/IV chemical activators were chosen in the present study rather than applying substances separately because previous studies found that the sum of stimuli, applied separately, induced a lower firing rate than the combination of stimuli (Light et al., 2008; Birdsong et al., 2010). Indeed, we have shown that the P2X and other molecular receptors, i.e., acid-sensing ion channel number 3 (ASIC3), located in the metabosensitive afferent endings are triggered by their specific metabolite, but could also interact with one another to potentiate their activities. Moreover, in our study, combination of KCl, LA, and ATP confirmed that metabosensitive group III/IV afferents were activated because their discharge was strongly decreased when P2X receptors were blocked by PPADS.

The dorsal root stimulation-evoked somatic reflex was considered to be more suitable than the Hoffman reflex that we have previously performed (Pin-Barre et al., 2014). Indeed, the dorsal root stimulation could depolarize the largest sensory axons to evoke the somatic reflex without depolarizing the motor axons at the same time. It thus avoided the generation of an antidromic wave, which partially blocks the information arising from the largest afferent pathways, thereby disturbing the reflex amplitude.

We also confirmed that stimulation of metabosensitive muscle afferents by chemical injections were responsible of somatic reflex changes (Pettorossi et al., 1999; Kalezic et al., 2004; Laurin et al., 2010, 2016): (1) when no injection was performed during reflex recordings or when saline (same volume, 0.4 mL) was injected, maximal amplitude of reflex wave was not modified and (2) when P2X receptors are blocked, the somatic reflex amplitude was no longer changed after the chemical injections. For the first time, it was shown that P2X receptors might influence spinal somatic reflex.

Other methodological points indicated that firing rate of VPL nuclei could be activated by information originating from the metabosensitive muscle afferents. First, no discharge increase was observed after saline injection. Then, the interruption of blood circulation in the brachial vein during recordings prevented the activation of VPL via humoral diffusion of chemical activators.

One limitation of this study was that we did not block the other receptors activating these muscle afferents such as ASICs (sensitive to LA concentration). Nevertheless, the molecular receptors triggered by metabolites acted in a synergetic way because detection of LA and ATP was made by interplay of two ion channels (ASICs and P2X) (Birdsong et al., 2010). Thus, the P2X blockade could affect the ASICs activity, and consequently, explained why the afferent discharge decreased following injections of our metabolite combinations.

In addition, the non-paretic side was not investigated in the present study because of the duration of electrophysiological recordings for each animal. Nevertheless, it is noteworthy to add that it should be interesting to compare the spinal reflex response between the affected and non-affected muscles to assess the spinal neuroplasticity on both sides. Indeed, divergent findings remain about the impact of fatigue at each side (Knorr et al., 2012).

We used intramuscular electrodes to record spinal reflex amplitude. The limitation is linked to the fact that muscle mechanical constraints induced by contraction could influence the amplitude of the reflex wave contrary to the recording on the efferent nerve (Türker, 1993). However, we also observed, in accordance with Hayashi et al. (2001), that it is not possible to reach the ventral root at the cervical spinal cord level with electrodes. Indeed, the ventral roots innervating the forelimb muscles are too short and too deep to place on electrodes. It explains why we have to choose intramuscular recording electrodes.

Moreover, we could not directly measure the concentration of ATP, LA, and KCl into the *triceps brachii* after the injection. Nevertheless, we performed a pre-experiment (data not shown) in which we injected methylene blue solution into the collateral artery and we observed that this product well reached this muscle (colored in blue after the injection). Moreover, the afferent receptors in muscle have been reported to be on the outside of blood vessels supplying muscle (Molliver et al., 2005). In our study, we thus knew that the product reach the *triceps brachii* blood vessels thanks to the administration of methylene blue solution.

### Changes in the Inhibitory Effect of Metabosensitive Afferents on Somatic Reflex Were Observed in Rats With MCAO

In the present study, the spinal plasticity resulting from cerebral ischemia is highlighted by an absence of somatic reflex inhibition following metabosensitive afferent activation. In line with our data, it was already observed a reduction in frequency-dependent depression of the H-reflex after traumatic brain injury (Tan et al., 2012). Moreover, our finding confirmed a previous study in which we postulated that these metabosensitive afferents might be involved in the reduction of H-reflex depression following fatiguing contraction (Pin-Barre et al., 2014). It was thus suggested that the disturbance of metabosensitive afferents effect on spinal reflex might partially explain the early fatigue observed during the acute phase of stroke. Indeed, an imbalance between excitatory and inhibitory activities might induce loss of muscle strength and coordination contributing to exaggerated muscular fatigue (Knorr et al., 2012; Phadke et al., 2012; Cho and Lee, 2013; Pin-Barre et al., 2014).

The disinhibition of somatic reflex could be caused by the disturbance of the afferent activity or by their effect at the spinal and/or supraspinal levels. In first place, we postulated that the molecular receptors might be impacted, thereby altering the discharge of group III/IV afferents because of the early muscular atrophy (Chang et al., 2010). However, the afferent discharge to KCl, LA, and ATP were not modified suggesting that molecular receptors were not disturbed throughout the first week (at D1 and D7). Nevertheless, it seems plausible that afferent receptor sensibility could be impaired in the chronic phase of the injury, as already proved (Wang et al., 2010). Moreover, given that the recording of group III and group IV afferent activity was not differentiated (because they are known to respond both to these activators), this might hide a different discharge pattern between group III and group IV afferents, as demonstrated in rats with myocardial infarction (Wang et al., 2010).

The above results suggested that the effect of group III/IV afferents on reflex pathway might be localized in the spinal cord at the level of the synaptic transmission between large diameter fibers (mainly group Ia afferents) and α-motoneurons. Several hypotheses might be highlighted to explain it, even though they need to be taken with caution. Although the spinal cord is a remote area of the cerebral ischemia, such result is not surprising because stroke-induced neuroinflammation, neuronal degeneration, and synaptic plasticity in the spinal cord from the first day that might contribute to sensorimotor deficits (Block et al., 2005; Hu et al., 2014; Tennant, 2014; Jones and Adkins, 2015). To reinforce this suggestion, it was demonstrated that brain injury induced maladaptive afferent fiber plasticity remote from the lesion into spinal cord gray matter regions and this was paralleled by increased microgliosis (Tan et al., 2012). In addition, it remained to determine if the reflex perturbation was originated from the pre- and/or post-synaptic levels.

### Activation of Metabosensitive Afferents Generated Bilateral VPL Nuclei Activities in Non-injured and Injured Animals

This study was the first to show in healthy rats that combinations of metabolites released during fatiguing contraction induced an increase of VPL firing rate on both sides. It reinforced previous data demonstrating that information from group III/IV afferents could induce an excitation of spinothalamic tracts (Foreman et al., 1979). We have first believed that metabosensitive afferents might induce a lateralized pattern of VPL activation (contralateral to the injection side) (Murphy and Corbett, 2009). Surprisingly, thalamic activation was mainly bilateral for healthy animals. Lateralized activation was only observed for the highest dose of activators, as reflected by higher contralateral VPL activation compared to the ipsilateral side. Given that no similar studies have been conducted at thalamic level to our knowledge, further studies are required to clarify the complex pattern of VPL activity when the metabosensitive afferents are activated.

After cerebral ischemia, the discharge of afferents also induced a bilateral activation, but no difference was observed compared with healthy animals. The high variability of VPL firing rate might explain similar results between groups. However, the bilateral pattern of activation was found for the three doses of activators in injured animals (i.e., including the highest dose) contrary to healthy animals. This result might indicate that thalamic response might be more bilateralized for animals with cerebral ischemia but such interpretation has to be taken with caution. Bilateral pattern of activation has already been observed following different types of sensory (mechanical) stimulations reflecting compensatory mechanism incapacities to restore lateralized nervous activities (Murphy and Corbett, 2009).

### The Sensorimotor Disorders of the Paretic Forepaw Were Maintained Throughout the Protocol

In accordance with previous studies (Andersen et al., 1991; Modo et al., 2000), MCAO induced both a reduction of mechanical sensibility and the motor coordination disorders, as reflected by a sustained increase of the time to detect and to remove the sticky label. Spontaneous progressive recovery was observed but remained incomplete at D7. This incomplete recovery seemed unlikely related to the evolution of cerebral infarct (no significant decrease between D1 and D7) which is in agreement with previous studies (Kleim et al., 2007; de Vries et al., 2011; Pin-Barre et al., 2014). Interestingly, the non-paretic forepaw also displayed sensibility disorders during 7 days. Motor deficits were identified but only during the 3 first days. These results were not surprising because bilateral functional perturbation was previously observed from 60 min of cerebral artery occlusion (Freret et al., 2006). It assumed that severe ischemia did not only cause damages in the ischemic tissues but also induce changes of CT hemisphere, thereby impacting sensorimotor functions of the non-paretic side (Block et al., 2005; Imbrosci et al., 2015).

The mechanical sensibility threshold to pressure was increased for the paretic forepaw confirming the loss of tactile sensibility. To our knowledge, no previous study measured mechanical sensibility to pressure with EVF test after 2 h of MCAO. These observations were in contradiction with previous works in which a reduced mechanical threshold to pressure was observed after global brain ischemia or focal ischemia of the thalamus, reflecting tactile hypersensibility and/or allodynia in neuropathic pain models (Matsuura et al., 2016a,b). Contrary to ART, the EVF test did not reveal mechanical sensibility disorders of the right forepaw. The different types of stimuli applied might explain this divergence of results because EVF test allowed measuring fine touch that is more localized than the sticky label application.

## Conclusion

This study is the first to provide evidence that the effect of group III/IV afferents on somatic reflex was changed after cerebral ischemia. Considering these afferents are involved in the regulation of motor adjustment during a muscular contraction, it might contribute to the observed behavioral disturbances during the acute phase of cerebral ischemia. Moreover, the plasticity observed in the thalamus should be more investigated due to its role in the integration of sensory feedback from muscles. This study suggested that spinal plasticity should not be overlooked when assessing the effectiveness of treatments on functional recovery.

## Author Contributions

JL and CP-B made substantial contributions to the conception of the project, performed the experiments, analysis, and interpretation of data, and wrote the manuscript. CP made substantial contributions to the conception of the project and participated in drafting the manuscript. CP contributed to the analysis and interpretation of data. FL made substantial contributions to the conception of the project, and developed all the MATLAB programs needed for processing data that change for us the way to perform electrophysiological recordings. FL participated in drafting the manuscript.

## Conflict of Interest Statement

The authors declare that the research was conducted in the absence of any commercial or financial relationships that could be construed as a potential conflict of interest.
